# Inhibition of AMPK/PFKFB3 mediated glycolysis synergizes with penfluridol to suppress gallbladder cancer growth

**DOI:** 10.1186/s12964-022-00882-8

**Published:** 2022-07-16

**Authors:** Jiahao Hu, Jiasheng Cao, Ren’an Jin, Bin Zhang, Win Topatana, Sarun Juengpanich, Shijie Li, Tian’en Chen, Ziyi Lu, Xiujun Cai, Mingyu Chen

**Affiliations:** 1 Department of General Surgery, Sir Run-Run Shaw Hospital, Zhejiang University, No. 3 East Qingchun Road, Hangzhou, 310016 Zhejiang Province China; 2 School of Medicine, Zhejiang University, Hangzhou, 310058 Zhejiang Provinc China; 3 Key Laboratory of Laparoscopic Technique Research of Zhejiang Province, No. 3 East Qingchun Road, Hangzhou, 310016 Zhejiang Province China; 4 Zhejiang Minimal Invasive Diagnosis and Treatment Technology Research Center of Severe Hepatobiliary Disease, Hangzhou, China; 5 Zhejiang Research and Development Engineering Laboratory of Minimally Invasive Technology and Equipment, Hangzhou, China

**Keywords:** Gallbladder cancer, Penfluridol, Apoptosis, Glycolysis, AMPK/PFKFB3

## Abstract

**Background:**

Penfluridol (PF) is an FDA-approved antipsychotic drug that has recently been shown to have anticancer activity. However, the anticancer effects and underlying mechanisms of PF are not well-established in gallbladder cancer (GBC).

**Methods:**

The anticancer efficacy of PF on GBC was investigated via a series of cell functions experiments, including cell viability, colony formation, apoptosis assays, and so on. The corresponding signaling changes after PF treatment were explored by western blotting. Then, nude mice were utilized to study and test the anticancer activity of PF in vivo. Besides, glucose consumption and lactic production assays were used to detect the glycolysis alteration.

**Results:**

In this study, we discovered that PF greatly inhibited the proliferation and invasion ability of GBC cells (GBCs). The glucose consumption and lactic generation ability of GBCs were dramatically elevated following PF treatment. Additionally, we discovered that inhibiting glycolysis could improve PF's anticancer efficacy. Further studies established that the activation of the AMPK/PFKFB3 signaling pathway medicated glycolysis after PF treatment. We proved mechanistically that inhibition of AMPK/PFKFB3 singling pathway mediated glycolysis was a potential synergetic strategy to improve the anticancer efficacy of PF on GBC.

**Conclusions:**

By inhibiting AMPK, the anticancer effects of PF on GBCs were amplified. As a result, our investigations shed new light on the possibility of repurposing PF as an anticancer drug for GBC, and AMPK inhibition in combination with PF may represent a novel therapeutic strategy for GBC.

**Graphical abstract:**

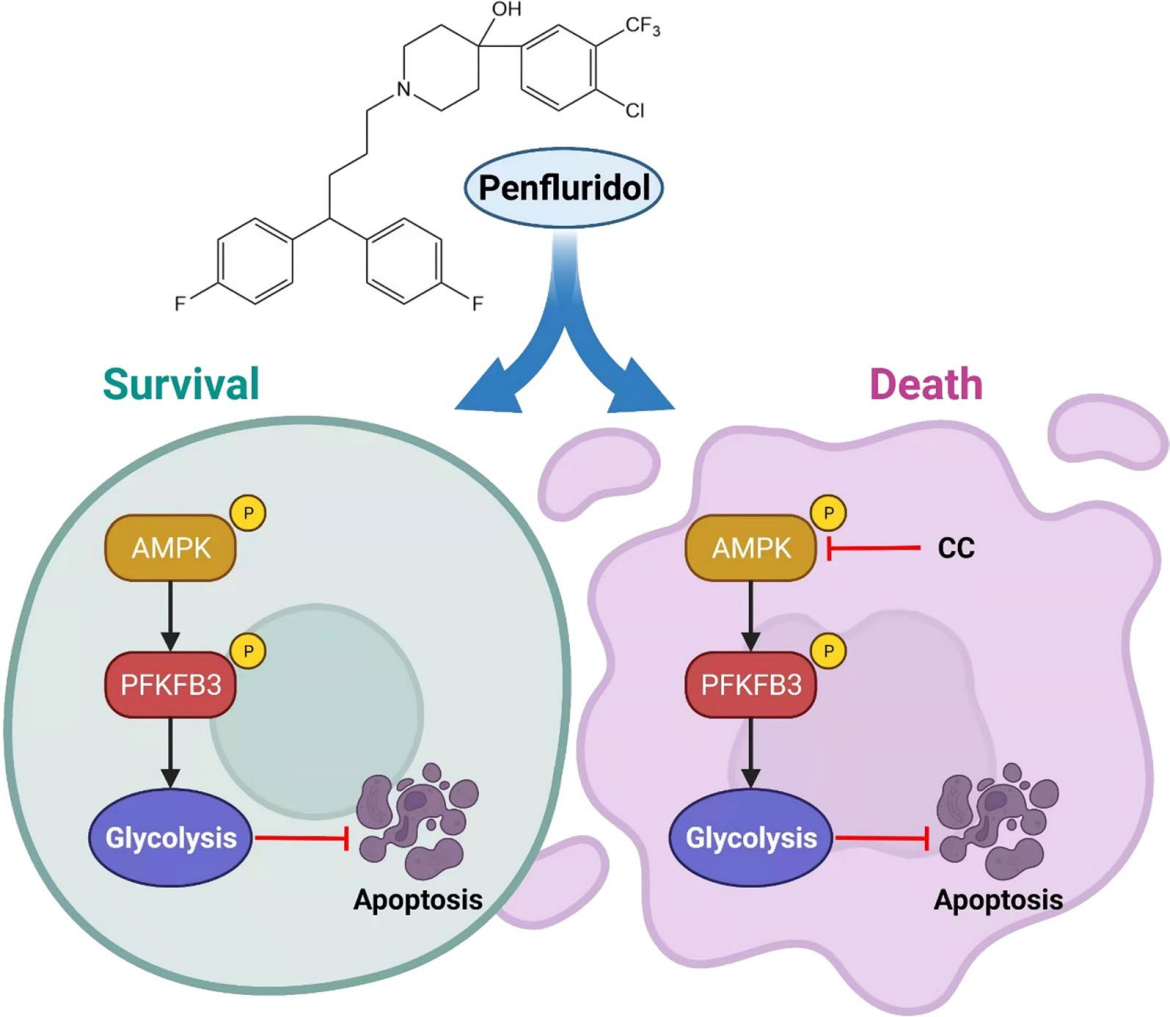

**Video abstract**

**Supplementary Information:**

The online version contains supplementary material available at 10.1186/s12964-022-00882-8.

## Background

Gallbladder cancers (GBC) is one of the most common biliary tract cancers, with poor prognosis and rising prevalence, particularly among American Indians and Southeast Asians [1, 2]. Surgical resection is the first-line treatment for patients with early GBC contributing to a near-perfect long-term survival. For patients diagnosed as unresectable or metastatic GBC, gemcitabine plus cisplatin is recommended as the primary treatment [3]. Unfortunately, adverse events are commonly observed and prognosis remains unsatisfactory after chemotherapy for advanced GBC [4]. Therefore, it is necessary to explore adequate treatments to improve prognosis with fewer side effects for GBC patients.

In recent years, several non-anticancer drugs have been repurposed for cancer treatment, such as metformin for colorectal cancer, and nitroxoline for pancreatic cancer [5, 6]. The rationale for repurposing antipsychotic medications for cancer therapy stemmed from multiple clinical studies, demonstrating that patients receiving antipsychotic medications had a lower cancer incidence [7]. Penfluridol (PF), a first-generation FDA-approved antipsychotic drug, exhibited anticancer activity [8]. PF affected several hallmark aspects of cancers, including tumor-promoting inflammation, immune evasion, activation invasion and metastasis, cell death resistance, and sustaining proliferative signaling [9]. While multiple anticancer mechanisms were reported, such as inhibiting the integrin signaling pathway in breast cancer and inducing autophagy to promote cancer apoptosis, the precise mechanism in GBC remains unclear [10, 11].

Reprogramming energy metabolism (Warburg Effect) has been recognized as a “hallmark of cancer” in malignancies [12, 13]. Even in aerobic conditions, cancer cells rely more on glycolysis to generate energy and metabolic intermediates for the maintenance of cancer biosynthesis, which was maintained by elevated glucose uptake [14, 15]. The tumor-specific metabolic changes may provide targets for therapeutic strategies. Patients who receive antipsychotic medication have a greater risk of developing metabolic syndrome, suggesting that anti-psychotics may affect metabolism [16]. For example, clozapine could promote glucose metabolism in oligodendrocytes [17]. However, the role of PF on glycolysis metabolism as well as the underlying mechanism for cancer therapy remains unclear and waiting for further exploration.

In this study, we repurposed the anticancer activity of PF on GBC. Additionally, we investigated whether activating the AMPK/PFKFB3 signaling pathway mediated glycolysis following PF treatment diminished PF's anticancer effects. Our findings indicated that combining PF with an inhibitor of its glycolytic adaptation (inhibiting AMPK) may be a viable strategy for treating gallbladder cancer.

## Methods

### Reagents and antibodies

The following reagents were used in the study: Penfluridol (PF), 2-Deoxy-D-glucose (2-DG), A-769662, Compound C (CC) were purchased from MedChemExpress (MCE, Shanghai, China), Cell Counting Kit-8 (CCK-8) were obtained from Yeasen (Shanghai, China), Annexin V/PI Apoptosis staining Kit, and cell cycle staining kit were purchased from MultiSciences (70-CCS012, Hangzhou, China), si-AMPK (5’- GGTTGGCAAACATGAATTG 3’) were obtained from Ribobio (Guangzhou, China), Glucose colorimetric assay kit, and L-Lactic Acid colorimetric assay kit were purchased from Elabscience (Wuhan, China), Lipofectamine 3000 reagent, and BCA protein assay kit were purchased from Invitrogen (CA, USA), 4% paraformaldehyde, 0.1% crystal violet, and RIPA were purchased from FUDE BIOLOGICAL (Hangzhou, China), ibidi Culture-insert was purchased from ibidi (Martinsried, Germany), transwell plate were purchased from Corning (Corning, NY, USA), human CA242 ELISA kit was purchased from Wuhan Fine Biotech (EH0398, Wuhan, China).

The anti-β-actin (FD0060), secondary antibodies goat-anti-rabbit (FD0128) and goat anti-mouse (FD0142) IgG-HRP antibody was purchased from FUDE BIOLOGICAL Technology (Hangzhou, China), Anti-Caspase-3 (ER30804), anti-activated caspase-3 (ET1602-47), anti-PARP (ET1608-56), anti-cleaved-PARP (ET1608-10) were purchased from HUABIO (Hangzhou China). Anti-AMPK (ab207442), and anti-PFKFB3 (ab181861) were purchased from Abcam (Cambridge, UK). Anti-PFKFB3^S461^ (TA3581), and anti-AMPK^T172^ (TP56027) were purchased from Abmart (Shanghai, China).

### Cell culture

Human GBC cell lines EH-GB1 and GBC-SD were purchased from a cell bank (Chinese Academy of Sciences, Shanghai, China). The SGC-996 cell line was provided by Dr. Ying-bin Liu’s lab at Xin Hua Hospital Affiliated with Shanghai Jiao Tong University School of Medicine, China. These cells were maintained in DMEM (EH-GB1 and GBC-SD) and RPMI-1640 (SGC-996) medium containing 10% FBS (cellMax, Beijing, China), penicillin (100 units/ml) and streptomycin (100 μg/ml) in a 5% CO2 humidified incubator at 37 °C (Thermo Scientific).

### Cell viability assay

Cell viability was assessed by CCK-8 kit according to the manufacturer’s instructions. Cells (3 × 10^3^) were seeded in 96-well plates and allowed to attach overnight in a 5% CO_2_ incubator. The cells were then treated with agents at indicated concentrations for 24 h or 48 h. The absorbance at 450 nm was measured with a multiscan spectrophotometer (Thermo Scientific).

### Colony formation assay

1 × 10^3^ cells/well were seeded in triplicate in a six-well plate for 24 h. Then, the original medium was replaced with medium-containing agents at each concentration tested. Cells were incubated for another 14 days and replaced with the culture medium at an appropriate time. Photographed after cells were fixed by 4% paraformaldehyde and stained with 0.1% crystal violet and counted.

### Wound-healing assay

GBC cells were pre-treated for 12 h as indicated. Then, cells were digested and resuspended with a culture medium. 2 × 10^4^ cells/well were seeded into ibidi Culture-insert on 12-well plates for 12 h. Later, the insert was gently removed, and the medium was replaced with an FBS-free medium. Photomicrographs were taken by a microscope (Zeiss, Germany) at the indicated time.

### Transwell migration assay

GBC cells were pre-treated for 12 h as indicated. Then, cells were digested and resuspended with an FBS-free medium. 2 × 10^4^ cells/well were seeded into the upper chamber of transwell 24-well plates with 8 μm pore size. Then, the lower chamber was added with a medium containing 20% FBS. After incubation of 24 h or 48 h, the upper surface of the chamber was cleaned, and migrated cells of the lower surface were fixed by 4% paraformaldehyde and stained with 0.1% crystal violet for 4 min. The level of migration was observed by a microscope.

### Detection of cell apoptosis

Cell apoptosis was detected by staining using Annexin V/PI Apoptosis staining Kit according to the manufacturer’s instructions. Briefly, GBC cells were digested with EDTA-free trypsin after agent treatment and resuspended in 500μL of binding buffer. After incubation with 5μL Annexin V-FITC and 10μL PI for 15 min at room temperature in the dark, data acquisition and analysis were carried out using flow cytometry.

### Detection of cell cycle

Cell cycle was detected by staining using a cell cycle staining kit according to the manufacturer’s instructions. Briefly, GBC cells were digested with EDTA-free trypsin after agent treatment and resuspended in 1 ml of DNA staining solution. After incubation with 10μL of Permeabilization solution for 30 min at room temperature in the dark, data acquisition and analysis were carried out using flow cytometry.

### Western blot

Cells were seeded into a six-well plate (80,000 cells/well) overnight and treated with indicated agents. After treatment, cells were collected by being scraped and washed with PBS. Then, the cells were lysed with RIPA buffer. Protein concentrations in the supernatants were determined with a BCA protein kit. Equal amounts of proteins were separated on SDS–polyacrylamide gels and then electroblotted onto polyvinylidene fluoride membranes (Sigma-Aldrich). The membrane was blocked with 5% skimmed milk in TBST for 1 h at room temperature and incubated with primary antibodies overnight at 4 °C. After being washed three times with TBST, the membrane was ncubated with secondary antibodies (1:5000) for 1 h at room temperature. Before detecting the target protein, the membrane was washed three times again, and the immunoblots were visualized with an ECL system.

### Glucose consumption measurement

Cell glucose consumption was detected by Glucose colorimetric assay kit according to the manufacturer’s instructions. Briefly, cells were planted in the six-well plates (80,000 cells/well) and incubated for 24 h. Then, the culture medium was replaced with a medium with different agents. After 24 h, the supernatant was collected and measured for glucose concentration. The cells left were lysed by RIPA for the detection of protein concentration to standardize glucose consumption levels.

### Lactic acid production measurement

Cell lactic acid production was detected by L-Lactic Acid colorimetric assay kit according to the manufacturer’s instructions. Briefly, cells were planted in the six-well plates (80,000 cells/well) and incubated for 24 h. Then, the culture medium was replaced with a medium with different agents. After 24 h, the supernatant was collected and measured for lactic acid concentration. The cells left were lysed by RIPA for the detection of protein concentration to standardize glucose consumption levels.

### Patient-derived xenograft (PDX) model establishment

Tumor tissues from the patient were evaluated by pathologists and macro-dissected for implantation. 4-week-old female nude mice were purchased from Hangzhou Ziyuan Experimental Animal Technology Co., Ltd (Hangzhou, China) and maintained in a pathogen-free environment. The mice were transplanted with tumor tissues subcutaneously. During the grafting, all mice were intact. Initial tumor growth was detected at 4 months post-implantation. The tumors were serially implanted into new female nude mice via the same procedure as the original implants. A line was considered established if there was active growth after at least five passages. Tumor samples were harvested from later passages (> 3) for characterization.

### In vivo study

Xenograft tumors were established by subcutaneous transplantation. After 10 days, to assess the anticancer effects of agents on tumor growth, tumor-bearing mice were randomly divided into four groups: the control group (10% DMSO, 40% polyethylene glycol, and 5% Tween-80 in saline), CC treatment group (10 mg/kg CC, intratumor injection, twice every week), PF treatment group (10 mg/kg, ig, qd), and CC plus PF treatment group. Tumor volumes were measured every 3 days and calculated by the followed equation: Volume = (Length × Width^2^)/2. On day15, mice were sacrificed for the tumor tissue collection.

### ELISA assay

Blood samples were collected from the retro-orbital bleed of nude mice to determine the serum CA242 level. The blood samples were centrifuged for 20 min (1000×*g*) at room temperature and retained the serum. The sample serum was diluted by sample dilution buffer at a fivefold dilution. After incubation at 37 °C for 90 min, the plate was washed by washing buffer twice and incubated at 37 °C with a 1:100 diluted biotin-labeled antibody at 100 μl/well. After incubation for 60 min, the plate was washed by washing buffer three times and incubated at 37 °C with a 1:100 diluted HPR-Streptavidin Conjugate solution at 100 μl/well. After incubated for 30 min, the plate was washed five times by washing buffer and incubated with TMB substrate solution at 100 μl/well at 37 °C in the dark for 10-20 min. After adding 50 μl/well of stop solution, the absorbance at 450 nm was measured immediately.

### Immunohistochemical staining

Tumor tissues were acquired and fixed overnight in 4% paraformaldehyde and then dehydrated and coated with wax. Paraffin-embedded specimens were cut into 3-μm thick sections. Slices were either dyed with hematoxylin and eosin or immunostained with the primary antibodies. DAB system was used to detect positive staining cells, and hematoxylin was used for counterstain.

### Statistical analysis

All experiments were performed at least three times in triplicate to ensure reproducibility. Statistical analyses used unpaired tailed student’s *t*-tests, Kaplan–Meier survival analysis, and log-rank tests with GraphPad Prism 7 (GraphPad Software, Inc., La Jolla, CA). The results were shown as mean ± SD. *P* < 0.05 was considered statistically significant.

## Results

### Penfluridol induced apoptosis, cell cycle arrest, and reduced migration ability of GBCs

**Fig. 1:**
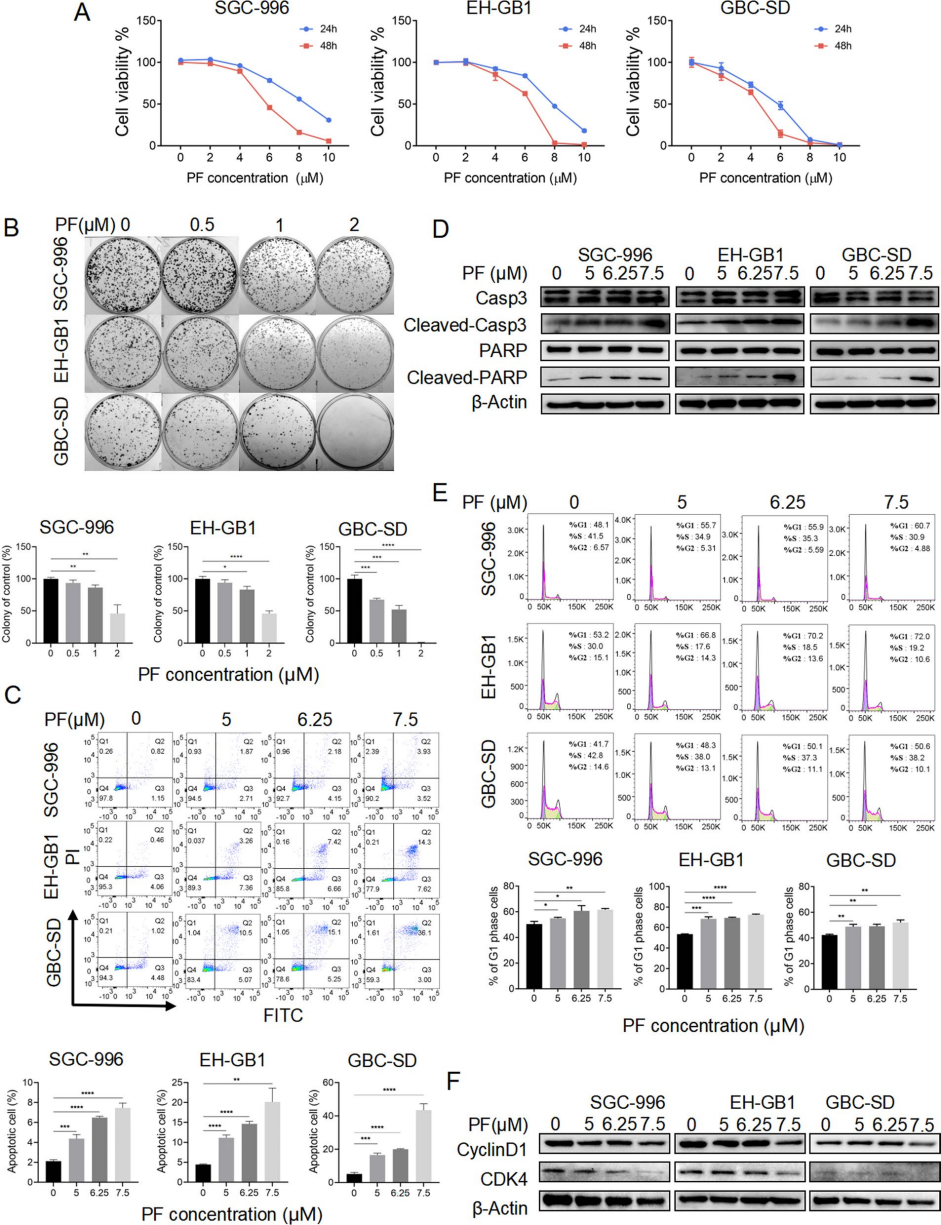
The anticancer effects of penfluridol on GBC. **A** The cell viability measured by the CCK-8 assay of SGC-996, EH-GB1, and GBC-SD cells were treated with PF as indicated for 24 h/48 h. **B** The colony formation of SGC-996, EH-GB1, and GBC-SD cells were seeded in 6-well plates and treated with PF as indicated for 14 days. **C** Annexin V/PI staining apoptosis assay of SGC-996, EH-GB1, and GBC-SD cells treated with PF as indicated for 24 h. **D** The Western blotting of SGC-996, EH-GB1, and GBC-SD were treated with PF as indicated for 24 h. **E** Cell cycle assay of SGC-996, EH-GB1, and GBC-SD cells treated with PF as indicated for 24 h. **F** The Western blotting of SGC-996, EH-GB1, and GBC-SD were treated with PF as indicated for 24 h. *P < 0.5, **P < 0.01, ***P < 0.001, ****P < 0.0001

To investigate the anticancer effects of PF on GBC cells (GBCs), we treated the GBCs (SGC-996, EH-GB1, and GBC-SD) with different concentrations of PF (2–10 μM). According to the results of the CCK-8 assay, the cytotoxicity of PF increased in a dose-dependent manner (Fig. 1A). The colony formation assay indicated that the low dosage of PF also suppressed the proliferation of GBCs (Fig. 1B). To identify whether PF treatment induces GBC cell death, a FACS analysis was performed by Annexin V-FITC/PI staining. PF treatment also induced dose-dependent apoptosis in GBCs (Fig. 1C). Furthermore, increased cleaved Caspase3 and PARP were detected in GBCs receiving PF treatment consisting with the FACS results (Fig. 1D). We next assessed the effect of PF on cell cycle distribution by flow cytometry analysis. As shown (Fig. 1E), PF treatment induced the accumulation of cells in the G1 phase. Cyclin D1 associated with cyclin-dependent kinase 4 was crucial for the transition from G1 to S phase [18]. Protein expression of cyclin D1 and CDK4 were decreased after PF treatment by western blotting analysis (Fig. 1F). Additionally, PF treatment also reduced the migration ability of GBCs by wound healing assay and transwell migration assay (Additional file 1: Fig. S1A, B). Taking together, PF showed anticancer effects on GBCs.

### Inhibiting glycolysis enhanced the anticancer effects of penfluridol

**Fig. 2:**
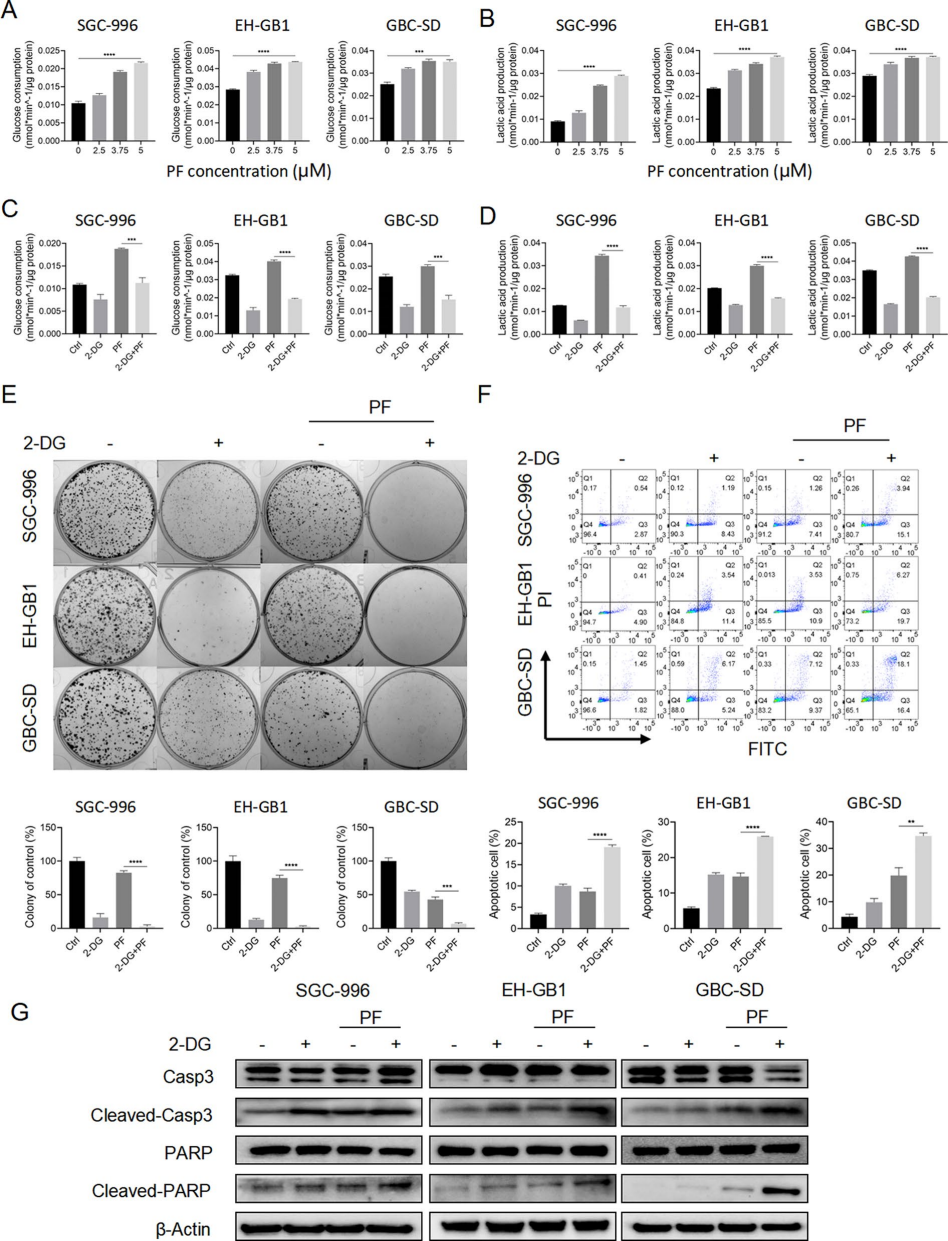
Inhibition of glycolysis enhanced the anticancer effects of penfluridol. **A**, **B** The glucose consumption and lactic ability of SGC-996, EH-GB1, and GBC-SD cells treated with PF as indicated for 24 h. **C**, **D** The glucose consumption and lactic ability of SGC-996, EH-GB1, and GBC-SD cells treated with control, 2-DG (2 mM), PF (5 μM), or 2-DG + PF. **E** The colony formation of SGC-996, EH-GB1, and GBC-SD cells treated with controls, 2-DG (1 mM), PF (1 μM), or 2-DG + PF for 14 days. **F** Annexin V/PI apoptosis assay of SGC-996, EH-GB1, and GBC-SD cells were with control, 2-DG (2 mM), PF (5 μM), or 2-DG + PF for 24 h before Annexin V/PI assay. **G** Western blot of SGC-996, EH-GB1, and GBC-SD were treated with control, 2-DG (2 mM), PF (5 μM), or 2-DG + PF. **P < 0.01, ***P < 0.001, ****P < 0.0001

During cell culture following PF treatment, we observed that GBCs treated with PF exhibited a more pronounced change in medium color (Additional file 2: Fig. S2A). This finding suggests that PF treatment may facilitate glycolysis in GBCs. As cancer cells generally rely on increased glycolysis even during aerobic conduction (Warburg effect), which is crucial for cancer cells to grow and even protects them from drug treatment [19]. Therefore, we hypothesize that the enhanced glycolysis is a cytoprotective strategy for GBCs to avoid death after PF treatment. We identified whether PF treatment promoted glycolysis via detecting glucose consumption and lactic acid production in GBCs. As expected, PF treatment increased glucose consumption and lactic acid production (Fig. 2A, B).

Then, we investigated the role of glycolysis in the anticancer efficacy of PF. Expectedly, the application of 2-DG, a glycolysis inhibitor, significantly reduced the glycolysis level after PF treatment (Fig. 2C, D). To determine the synergetic cytotoxic interaction of 2-DG and PF for GBCs, cell viability assay, colony formation assay, and Annexin V/PI apoptosis analysis were used (Fig. 2E, F and Additional file 2: Fig. S2B). Combining 2-DG with PF showed increased antitumor effectiveness as compared to monotherapy. Additionally, we also performed wound healing assay and transwell migration assay and found that combining 2-DG with PF suppressed migration abilities of GBCs more significantly compared to PF treatment alone (Additional file 2: Fig. S2C, D). These results suggested that promoted glycolysis may be a cytoprotective process in GBCs in response to PF treatment, and inhibiting glycolysis was an approach to improve the anticancer efficacy of PF in GBCs.

### Activation of AMPK/PFKFB3 mediated the glycolysis after penfluridol treatment

**Fig. 3:**
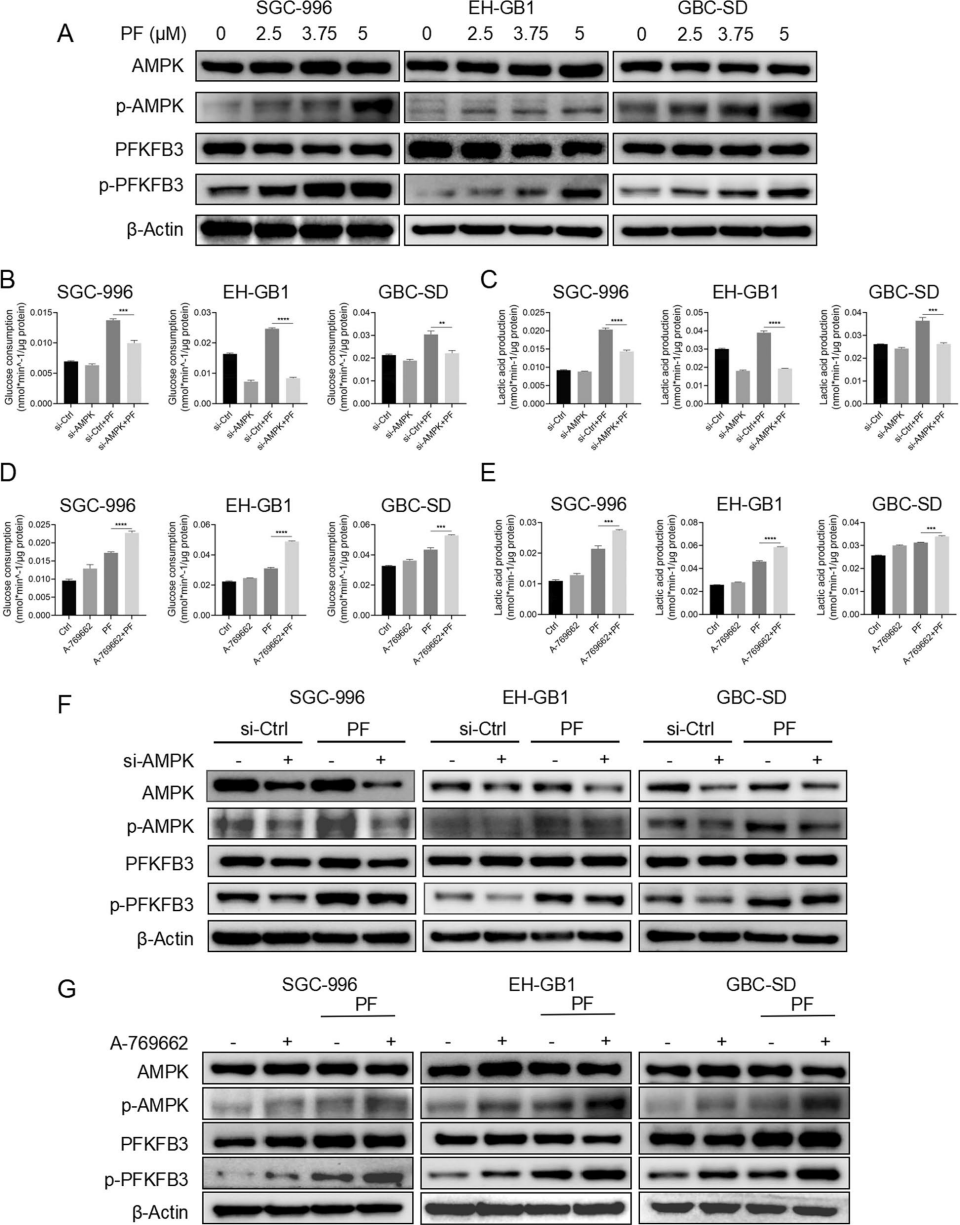
Activation of AMPK/PFKFB3 signaling pathway mediated the glycolysis after penfluridol treatment. **A** The Western blot of SGC-996, EH-GB1, and GBC-SD cells treated with PF as indicated for 24 h. **B**, **C** The glucose consumption and lactic acid production ability of SGC-996, EH-GB1, and GBC-SD cells treated with control and PF (5 μM) in the presence or absence of AMPK si-RNA. **D**, **E** The glucose consumption and lactic acid production ability of SGC-996, EH-GB1, and GBC-SD cells treated with control and PF (5 μM) in the presence or absence of A-769662 (100 μM). **F** The Western blot of SGC-996, EH-GB1, and GBC-SD cells treated with control and PF (5 μM) in the presence or absence of AMPK si-RNA. **G** The Western blot of SGC-996, EH-GB1, and GBC-SD cells treated with control and PF (5 μM) in the presence or absence of A-769662 (100 μM). **P < 0.01, ***P < 0.001, ****P < 0.0001

AMPK is a crucial energy sensor to monitor the cellular energy status, which could restore energy homeostasis via promoting catabolic pathways including glycolysis to generate ATP for cell growth [20]. PFKFB3, a bifunctional enzyme that is critical to the regulation of glycolytic flux, is downstream of AMPK [21]. To test whether PF treatment could activate AMPK as well as its downstream PFKFB3, GBCs (SGC-996, EH-GB1, and GBC-SD) were treated with indicated concentrations of PF. As shown (Fig. 3A), PF treatments were accompanied by increased phosphorylation of AMPK^Thr172^ and PFKFB3^Ser461^, indicating the activation of AMPK/PFKFB3 signaling [22].

To further identify whether AMPK was the central regulator in the PF therapy-induced glycolysis, we treated GBCs (SGC-996, EH-GB1, and GBC-SD) with 5 μM PF for 24 h in the presence or absence of siRNA-targeting AMPK or A-769662 (an AMPK activator). The glucose consumption and lactic acid assay elucidated that knockdown of AMPK suppressed the PF treatment-induced glycolysis as well as activation of AMPK improved PF treatment-induced glycolysis (Fig. 3B–E). Moreover, Western blot examination of cellular protein extracts revealed that reducing AMPK expression inhibited the PF treatment-induced increases in activating AMPK/PFKFB3 signaling and that activating AMPK with A-769662 synergized with PF treatment to phosphorylate AMPK and PFKFB3 (Fig. 3F, G). Our results demonstrated that AMPK/PFKFB3 mediated glycolysis was the central pathway to promote glycolysis after PF therapy.

### AMPK inhibitor compound C enhanced the anticancer effects of penfluridol

**Fig. 4:**
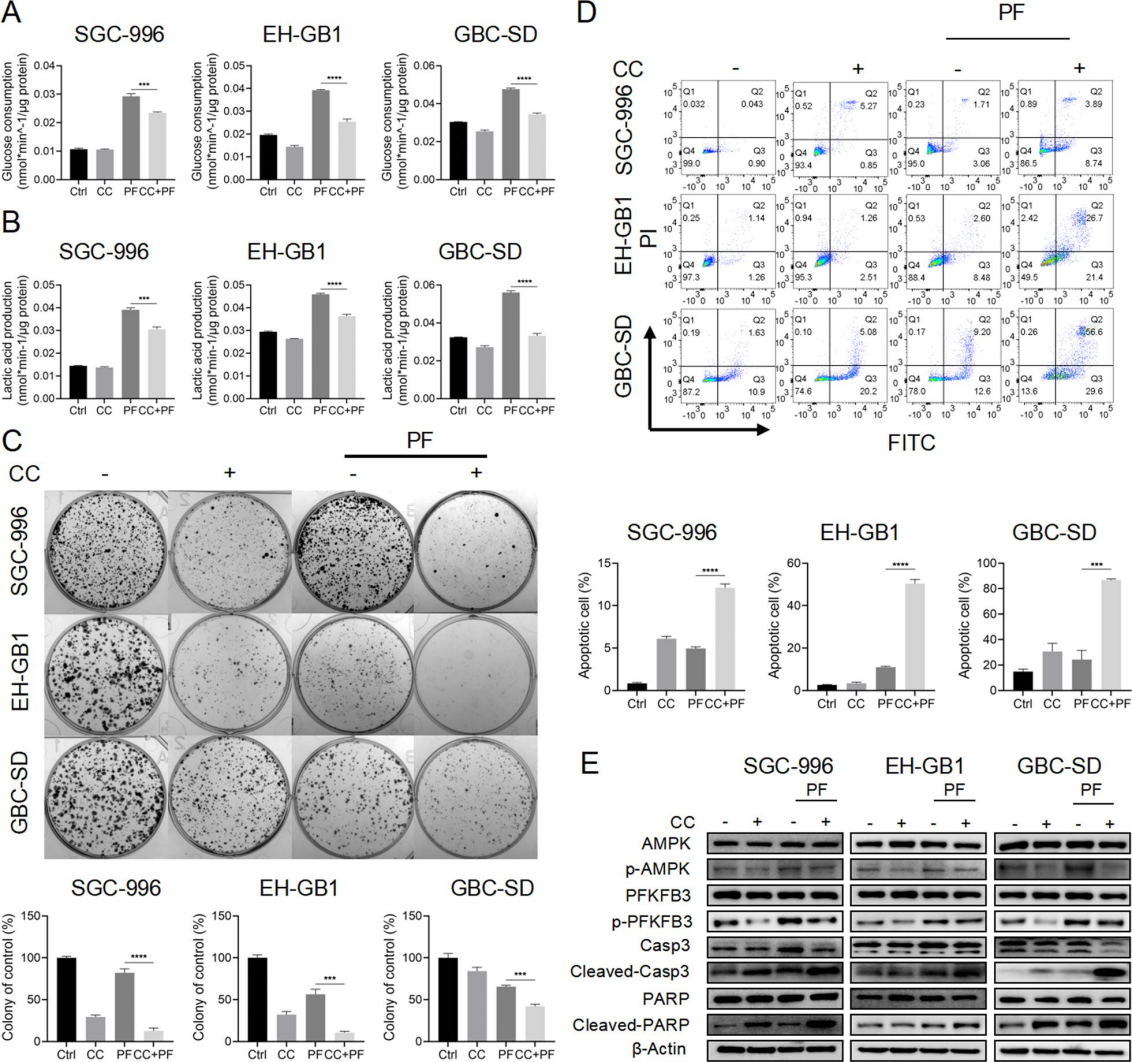
AMPK inhibitor CC enhanced the anticancer effects of penfluridol. **A**, **B** The glucose consumption and lactic acid production ability of SGC-996, EH-GB1, and GBC-SD cells treated with control, 10 μM CC, 5 μM PF, and CC + PF. **C** The colony formation of SGC-996, EH-GB1, and GBC-SD cells were treated with control, 10 μM CC, 5 μM PF, and CC + PF for 14 days. **D** The Annexin V/PI staining apoptosis assay of SGC-996, EH-GB1, and GBC-SD cells treated with control, 10 μM CC, 5 μM PF, and CC + PF. **E** The Western blot of SGC-996, EH-GB1, and GBC-SD cells treated with control, 10 μM CC, 5 μM PF, and CC + PF. ***P < 0.001, ****P < 0.0001

Given that inhibition of the AMPK/PFKFB3 pathway decreased glycolytic activity following PF treatment, it was reasonable to assume that inhibition of this pathway had a synergetic cytotoxic impact in GBCs in combination with PF. We firstly verified that the application of CC can inhibit PF therapy-induced glycolysis (Fig. 4A, B). Then, cell viability of SGC-996, EH-GB1, and GBC-SD was evaluated upon treatment with PF alone or in combination with CC. The results showed that when co-treated with CC, GBCs were more sensitive to PF therapy (Additional file 3: Fig. S3A). Colony formation assay suggested that the combination of Compound C and PF showed more significant cell death compared to monotherapy (Fig. 4C). Annexin V/PI apoptosis analysis further supported that PF treatment-induced apoptosis was significantly enhanced by CC in GBCs (Fig. 4D). Consistently, cleavage of Caspase 3 and PARP was found to be significantly increased in GBCs with Compound C/PF co-treatment in contrast to Compound C or PF treatment alone (Fig. 4E). Co-treated with PF and Compound C also had more significant inhibit effects on the migration ability of GBCs (Additional file 3: Fig. S3AB-C). Our results indicated that AMPK inhibitor CC synergized with PF to kill GBCs.

### Penfluridol in combination with Compound C effectively suppress the tumor xenografts in vivo

It is imperative to perform in vivo experiments to investigate the feasibility of the combination regimen of PF plus CC for the treatment of GBC. As PDX models retain the majority of the pathohistological and genetic characteristics of cancer and exhibit a more accurate response to therapeutic drugs, we created a GBC PDX model for further in vivo study[23]. Prior to conducting in vivo experiments, we identified the validity of our GBC PDX model. CA242, a sialic acid-containing carbohydrate antigen, is a fairly good marker for the diagnosis of GBC [24]. The serum CA242 levels in nude mice were determined using an ELISA assay. As expected, serum CA242 levels in nude mice were significantly higher in the GBC PDX model than in intact nude mice (Additional file 4: Fig. S4A). Additionally, the histological features of the xenografts from PDX models were high matched with the patient tumor (Additional file 4: Fig. S4B; CK7, CK19: positive; P40, P63: partial positive). The PDX model was then used to investigate the anticancer effects of PF and CC in vivo.

**Fig. 5:**
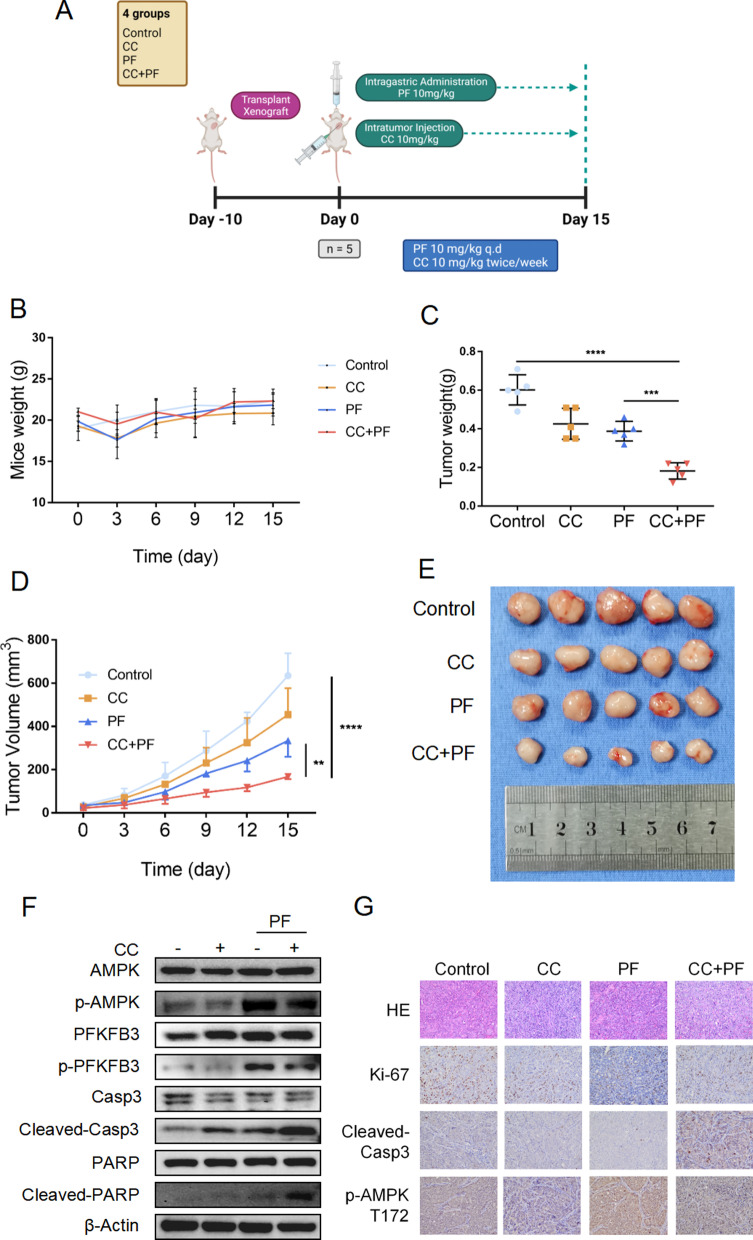
Penfluridol in combination with CC effectively suppresses the tumor xenografts in vivo. **A** The working model shows the process of our animal studies. Mice were transplanted with tumor tissues for 10 days and randomly divided into four groups (control, CC (10 mg/kg), PF (10 mg/kg), CC + PF; n = 5/group). Then, the tumor-bearing mice were treated with intragastric administration and intratumor injection with a dissolvent, intratumor injection with CC, intragastric administration with PF, and CC + PF co-treatment. All mice were sacrificed on day 15, and the tumors were collected for subsequent experiments. **B** The body weight change of mice after indicated treatment. **C** Tumor weight of the mice. **D** Tumor growth curves of tumor tissues after indicated treatment. **E** Photograph of the transplanted tumors after indicated treatment. **F** Western blotting assay result of the tumors with indicated treatment. **G** The H&E results and IHC staining assay for Ki-67, Cleaved-caspase3, p-AMPK T172 of the tumors. **P < 0.01, ****P < 0.0001

After ten days of tumor tissue transplantation, nude mice were randomly divided into four groups (n = 5): control, CC, PF, and CC plus PF. The tumor-bearing mice were treated as aforementioned (Fig. 5A). The weights of mice in treatment groups decreased at the beginning of the treatment and reached normal weight compared to the mice in the control group (Fig. 5B). Treatment with CC or PF alone both inhibited tumor growth, while CC plus PF combination treatment decreased tumor weights more significantly (Fig. 5C). The tumor growth curve data also indicated the potent anticancer effects of these combination regimens (Fig. 5D, E).

To examine the effects of CC and PF treatment on apoptosis and the activation of AMPK/PFKFB3 signaling in vivo, Western blot was performed. As shown in Fig. 5F, a significant increase of cleavage of Caspase 3 and PARP was detected in the CC plus PF group compared to the monotherapy group. Meanwhile, phosphorylation of AMPK was suppressed by CC after PF therapy. Lower Ki67 and higher cleavage Caspase 3 were induced by CC and PF, and, more significantly by CC and PF co-treatment (Fig. 5G).

## Discussion

We have demonstrated that PF, a first-generation antipsychotic, has anticancer effects in GBCs and that AMPK/PFKFB3 signaling-regulated glycolysis is the metabolic adaption mechanism used to mitigate PF-induced cytotoxicity. Additionally, we found that co-treatment with PF and CC can inhibit PF-induced glycolysis and improve PF's cytotoxicity in GBCs. Our data supported the combination drug regimen of PF plus CC for the treatment of GBC via the PDX model. Our results reveal a metabolic adaptation of AMPK/PFKFB3 signaling mediated glycolysis is an important pro-survival pathway in GBCs to protect them from the cytotoxicity of anticancer drugs and provide a theoretical basis of repurposing PF as an anticancer drug to GBC.

The anticancer mechanism of PF has been proposed by previous studies [9, 10, 25–27]. D_2_ receptor and T-type channel were major antipsychotic-related targets of PF to suppress cancer proliferation [28, 29]. Other signaling pathways such as the integrin signaling pathway and autophagy pathway were also reported in the anticancer mechanism of PF. However, there is little study reported to investigate the PF therapy-induced glycolytic adaptation and targeting the glycolysis in response to PF therapy to develop a therapeutic strategy. The therapeutic dose of PF for psychotic disorders is between 40 and 80 mg/week [30]. In our study, we administrated 0.2 mg/day of PF in a subcutaneous GBC PDX model. This dosage is the dose equivalent to about 75 mg/day in a 70 kg adult, which is significantly higher than its dosage for psychotic disease. Thus, inhibiting adaptive pathways may be a viable strategy for developing a more effective regimen strategy when considering the PF dosage for use as an anticancer agent.

Altered energy metabolism, also known as the Warburg effects, is one of the “hallmarks of cancer”, which supports the rapid cancer cells proliferation by preferential dependence on glycolysis even in the aerobic environment [12]. Apart from promoting tumorigenesis, some studies revealed that the Warburg effect could influence the anticancer efficacy of drugs. For example, increased GLUT3 is associated with temozolomide resistance in glioblastoma, and targeting GLUT3 could delay the acquired temozolomide resistance [31]. In our in vitro study, we found that enhanced glycolysis is a crucial adaptative response in GBCs to overcoming the cytotoxicity of PF therapy, and inhibiting glycolysis by 2-DG showed synergy effect with PF. However, directly inhibiting glycolytic pathways is difficult in the clinic due to undesirable effects such as systemic toxicity. Additionally, for the glycolytic inhibitor 2-DG we used, it was reported to activate the pro-survival pathway through IGF1R signaling [32]. Thus, further investigation of directly inhibiting glycolysis associated with PF therapy is necessary to assess whether 2-DG activates tumor-promoting pathways in GBC.

AMPK is a highly conserved energy regulator that plays a crucial role in maintaining energy homeostasis [33]. Previous studies suggested that activation of AMPK might suppress tumor growth, and AMPK activator metformin showed a tumor-suppressive effect in patients with diabetes [34]. However, it is indistinguishable whether its anticancer effects required AMPK. Recent studies suggested that AMPK showed a pro-survival effect for cancer cells in the circumstance of stressors such as growth factors and oncogenic stress [35]. The energy sensor AMPK conditional endows cancer cells survival advantages under selecting conditions to promote cancer cell proliferation and it is invertible when the extracellular environment is in favor of cancer progression [35]. Thus, it is logical that AMPK endows the survival of GBCs in response to anticancer treatments. In our present study, we found that AMPK activation induced by PF therapy promoted glycolysis via activating PFKFB3. AMPK inhibitor significantly suppressed the glycolysis levels after PF therapy and synergistically enhance the anticancer efficiency of PF in GBCs.

There were several limitations to be addressed in the study. Firstly, although we developed the GBC PDX model to assess the anticancer effect and safety of PF plus CC in vivo, the dosage of PF as an anticancer agent is still significantly higher than the clinically recommended dose, which may lead to severe side effects for patients. Thus, additional researches are needed to determine whether CC also synergizes with a lower PF dose in GBC. Second, in addition to regulating glycolysis, AMPK was involved in a variety of pathways involved in maintaining energy homeostasis. Additional research is needed to determine which other AMPK-regulated pathways are regulated in response to PF therapy, as well as to utilize PF's other adaptative mechanisms in order to minimize its dosage. Finally, further understandings of the underlying anticancer mechanism of PF are also needed to improve the anticancer efficacy of PF in GBC.

In this study, we demonstrated that PF could induce apoptosis to effectively suppress GBC cells proliferation. Moreover, activation of AMPK/PFKFB3-mediated glycolysis attenuated PF response in GBCs. The AMPK inhibitor CC suppressed the AMPK/PFKFB3 mediated glycolysis, which enhanced the anticancer effects of PF compared to PF monotherapy in GBCs. The synergetic effects of AMPK inhibition and PF were further validated in a PDX model.

## Conclusion

In summary, our study repurposed PF's anticancer properties for GBCs, and developed a combining regimen to improve the anticancer effect of PF by suppressing its AMPK/PFKFB3 mediated glycolytic adaptation. As a result, the combination of PF and CC could be used as anticancer strategy for GBC.

## Supplementary Information

**Additional file 1**. The anti-tumor effect of penfluridol on GBCs.

**Additional file 2**. Inhibition of glycolysis enhanced the anti-tumor effect of penfluridol.

**Additional file 3**. AMPK inhibitor CC enhanced the anti-tumor effect of penfluridol.

**Additional file 4**. Verifying the GBC PDX model.

## Data Availability

The data used during the current study are available from the corresponding author on reasonable request.

## Abbreviations

PF: Penfluridol
GBC: Gallbladder cancer
GBCs: GBC cells
CCK-8: Cell Counting Kit-8
2-DG: 2-Deoxy-D-glucose
CC: Compound C
