# Supplementary material for: Inhibition of AMPK/PFKFB3 mediated glycolysis synergizes with penfluridol to suppress gallbladder cancer growth
Source: Cell Commun Signal. 2022 Jul 16;20:105. doi: 10.1186/s12964-022-00882-8 (PMC9288071; doi:10.1186/s12964-022-00882-8)
Supplement: Supplementary file 2 — Additional file 1. The anti-tumor effect of penfluridol on GBCs. [file 12964_2022_882_MOESM2_ESM.docx]

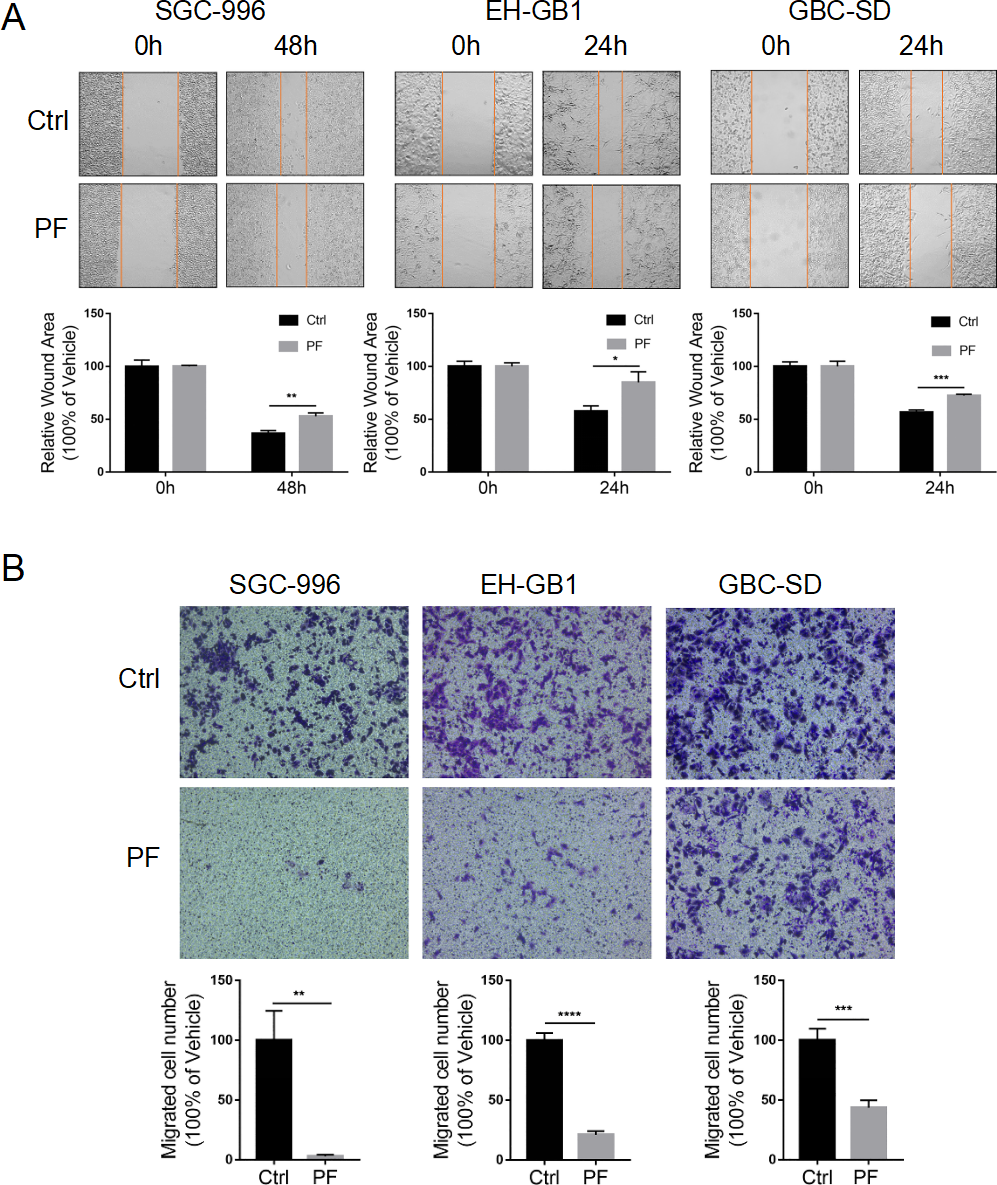


**Fig. S1** **The anti-tumor effect of penfluridol on GBCs. A** SGC-996, EH-GB1, and GBC-SD cells were pre-treated with 7.5μM PF for 12h before seeding into ibidi culture-insert. Wound healing was detected at indicated time after removing insert. **B** SGC-996, EH-GB1, and GBC-SD cells were pre-treated with 7.5μM PF for 12h before seeding into Transwell insert. Cellular migration was measured after 48h (for SGC-996) and 24h (for Eh-GB1 and GBC-SD). **P*<0.05, ***P*<0.01, ****P*<0.001, *****P*<0.0001.
