# Supplementary material for: Inhibition of AMPK/PFKFB3 mediated glycolysis synergizes with penfluridol to suppress gallbladder cancer growth
Source: Cell Commun Signal. 2022 Jul 16;20:105. doi: 10.1186/s12964-022-00882-8 (PMC9288071; doi:10.1186/s12964-022-00882-8)
Supplement: Supplementary file 3 — Additional file 2. Inhibition of glycolysis enhanced the anti-tumor effect of penfluridol. [file 12964_2022_882_MOESM3_ESM.docx]

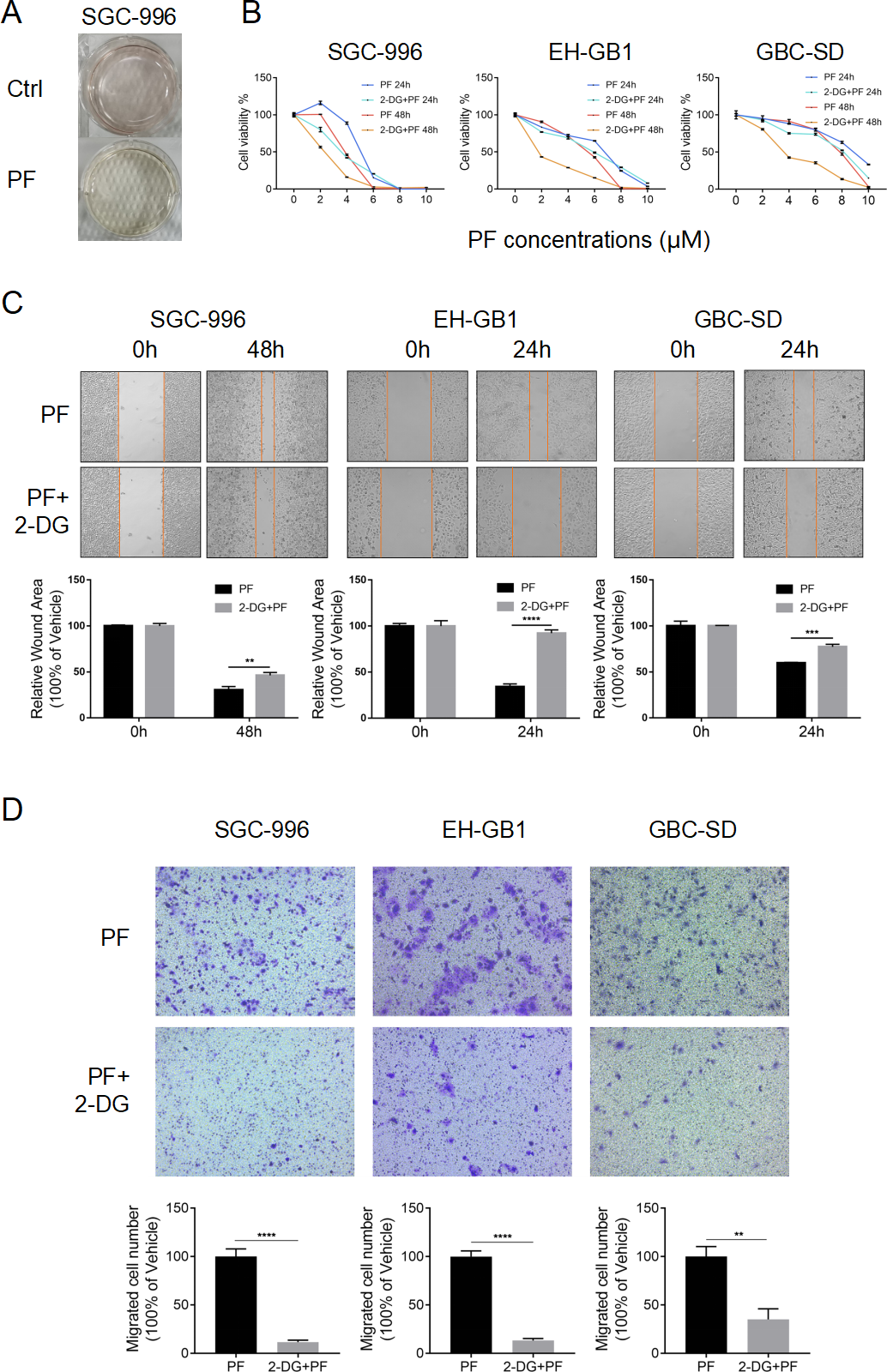


**Fig. S2** **Inhibition of glycolysis enhanced the anti-tumor effect of penfluridol.** **A.** The medium of SGC-996 after PD therapy. **B.** SGC-996, EH-GB1, and GBC-SD cells were treated with PF as indicated with or without 2-DG (2mM) for 24h/48h. Cell viability was measured by the CCK-8 assay. **C.** SGC-996, EH-GB1, and GBC-SD cells were pre-treated with 5μM PF with or without 2-DG (2mM) for 12h before seeding into ibidi culture-insert. Wound healing was detected at indicated time after removing insert. **D.** SGC-996, EH-GB1, and GBC-SD cells were pre-treated with 5μM PF with or without 2-DG (2mM) for 12h before seeding into Transwell insert. Cellular migration was measured after 48h (for SGC-996) and 24h (for Eh-GB1 and GBC-SD). ***P*<0.01, ****P*<0.001, *****P*<0.0001.
