# Supplementary material for: Inhibition of AMPK/PFKFB3 mediated glycolysis synergizes with penfluridol to suppress gallbladder cancer growth
Source: Cell Commun Signal. 2022 Jul 16;20:105. doi: 10.1186/s12964-022-00882-8 (PMC9288071; doi:10.1186/s12964-022-00882-8)
Supplement: Supplementary file 5 — Additional file 4. Verifying the GBC PDX model. [file 12964_2022_882_MOESM5_ESM.docx]

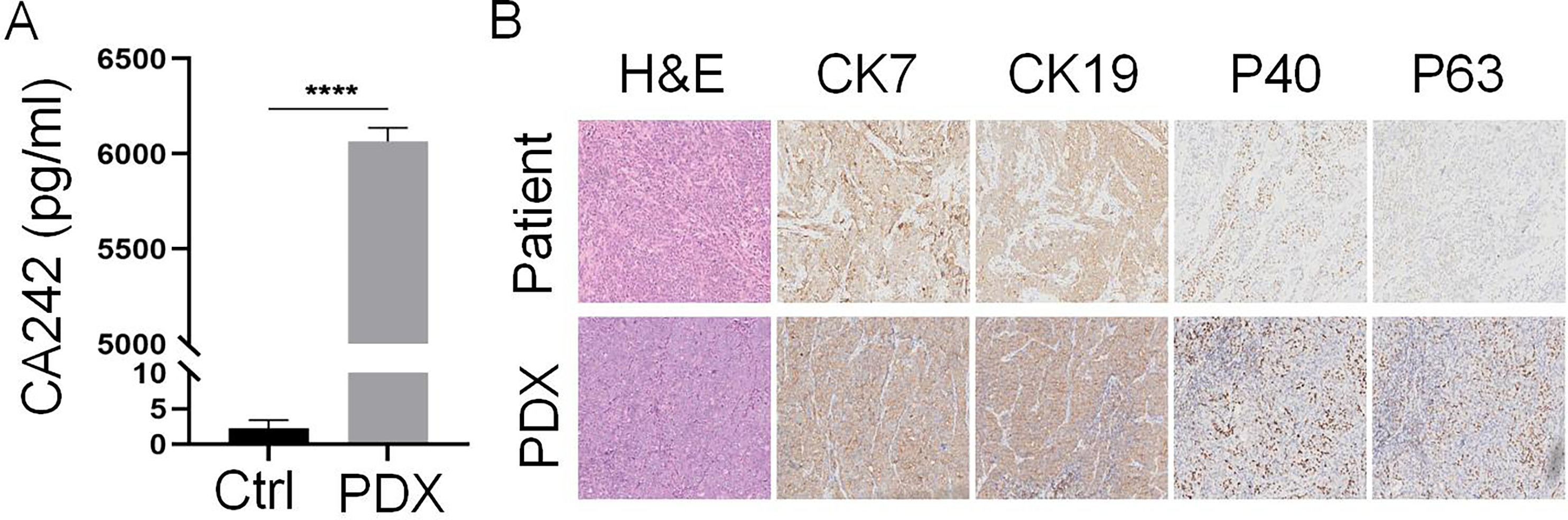


**Fig. S4** **Verifying the GBC PDX model.** **A.** The ELISA assay of human CA242 level in the serum of intact node mice and PDX model. **B.** The H&E-staining and the positive IHC staining results of the patient- and xenograft tumors.
